# Heterogeneous impacts of ocean thermal forcing on ice discharge from Greenland's peripheral tidewater glaciers over 2000–2021

**DOI:** 10.1038/s41598-024-61930-6

**Published:** 2024-05-17

**Authors:** Marco Möller, Beatriz Recinos, Philipp Rastner, Ben Marzeion

**Affiliations:** 1 Institute of Geography, University of Bremen, Bremen, Germany; 2 MARUM—Center for Marine Environmental Sciences, University of Bremen, Bremen, Germany; 3 Geodesy and Glaciology, Bavarian Academy of Sciences and Humanities, Munich, Germany; 4 School of GeoSciences, University of Edinburgh, Edinburgh, Scotland, UK; 5 Department of Geography, University of Zurich, Zurich, Switzerland

**Keywords:** Cryospheric science, Physical oceanography

## Abstract

The Greenland Ice Sheet is losing mass at increasing rates. Substantial amounts of this mass loss occur by ice discharge which is influenced by ocean thermal forcing. The ice sheet is surrounded by thousands of peripheral, dynamically decoupled glaciers. The mass loss from these glaciers is disproportionately high considering their negligible share in Greenland’ overall ice mass. We study the relevance of ocean thermal forcing for ice discharge evolution in the context of this contrasting behaviour. Our estimate of ice discharge from the peripheral tidewater glaciers yields a rather stable Greenland-wide mean of 5.40 ± 3.54 Gt a^−1^ over 2000–2021. The evolutions of ice discharge and ocean thermal forcing are heterogeneous around Greenland. We observe a significant sector-wide increase of ice discharge in the East and a significant sector-wide decrease in the Northeast. Ocean thermal forcing shows significant increases along the northern/eastern coast, while otherwise unchanged conditions or decreases prevail. For East Greenland, this implies a clear influence of ocean thermal forcing on ice discharge. Similarly, we find clear influences at peripheral tidewater glaciers with thick termini that are similar to ice sheet outlet glaciers. At the peripheral glaciers in Northeast Greenland ice discharge evolution opposes ocean thermal forcing for unknown reasons.

## Introduction

Ice mass loss of the Greenland ice sheet (GIS) has accelerated over recent decades, considerably impacting sea-level rise (SLR)^1,2^ and ocean circulation^3,4^. It increased sixfold since the 1980s^5^, reaching a new record of 532 ± 58 Gt a^−1^ in 2019^6^. Accordingly, its contribution to SLR increased from less than 5% to more than 25% during 1993–2014^7^. Ice discharge from numerous marine-terminating glaciers plays a key role in this development. It increased by 18 ± 1% between 1972–2000 and 2010–2018^5^. While in 2000–2012, about one third of the ice mass loss of the GIS was due to ice discharge, this share increased to more than half after 2013^8^. This substantial increase of ice discharge has generally been attributed to widespread ocean warming around Greenland^9–13^. Such a warming destabilizes grounding lines by increasing sub-marine melting and reduces buttressing effects by reduction of sea ice or ice melange concentration at the marine-terminating glacier fronts, leading to accelerated ice flow^14–16^. However, the ice discharge increases have continued after a widespread transition to subsurface ocean cooling in 2008^13,17^ and accordingly recent studies challenged an ongoing exclusive role of ocean thermal forcing in ice discharge evolution^18–20^, suggesting additional influencing factors or modifications of the governing processes on the local scale due to varying fjord geometries^21^. The GIS is not the only ice mass on Greenland. It is surrounded by more than 19,000 peripheral glaciers that are separated, or at least dynamically decoupled, from the GIS and cover more than 89,000 km^2^^22,23^. Almost one third of this area is contributed by a comparatively small number of 756 peripheral tidewater glaciers^23^ (PTG). Overall, the peripheral glaciers store an ice mass equivalent to 33.6 ± 8.7 mm SLR, which is two orders of magnitude smaller than the total ice mass of Greenland^24,25^. Despite this negligible share in total ice mass, the share of the peripheral glaciers in the total ice mass loss of Greenland was disproportionally high in the recent past. Total ice mass loss from the peripheral glaciers in the last two decades was suggested to lie in the range 27.9 ± 10.7–51 ± 17 Gt a^−1^, depending on the exact period and the study^26–30^. It was thus only one order of magnitude smaller than mass loss estimates reported for the GIS^5,6,8,31^.

Differing from the situation at the GIS, the peripheral glaciers did not show clear signs of mass loss acceleration after 2000^30^, and little is known about the partitioning of this loss in its surface and marine components. Overall, the peripheral glaciers and especially the PTG have shown considerable retreat over the twentieth century^32^ and afterwards^33^, and modelling studies found a negative trend of peripheral glacier-wide surface mass balance over the last three decades^34,35^. The related increase of surface mass loss suggests a decrease of ice discharge as compensation in order to arrive at the rather stable rates of total ice mass change^30^. This is in line with the limited knowledge about ice discharge from the PTG, which is provided by only three studies so far: while the absolute ice discharge estimates differ considerably, these studies agree on a general decrease of annual rates since 2000. The lower bound estimate suggests an ice discharge of 2.25 ± 0.77 Gt a^−1^ in 2000–2010 and 1.88 ± 0.43 Gt a^−1^ in 2010–2020^36^, while the upper bound more than doubles this estimate with 5.10 ± 0.21 Gt a^−1^ in 1999–2018^37^. The most recent study mediates between these two and comes up with an ice discharge of 3.15 ± 7.4 Gt a^−1^ in 2000–2010 and 2.31 ± 4.6 Gt a^−1^ in 2010–2020^38^.

**Figure 1:**
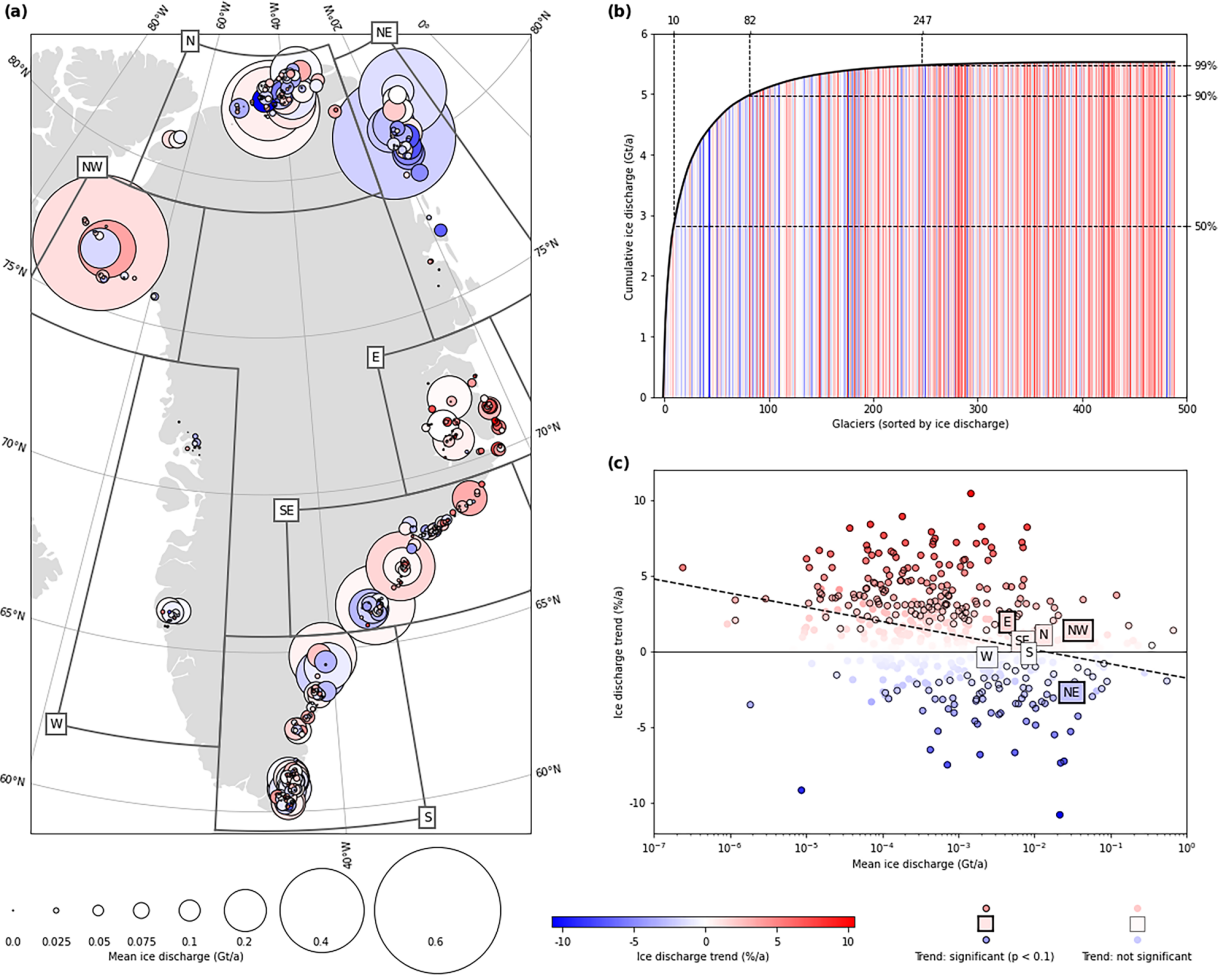
Ice discharge at the 489 peripheral tidewater glaciers of Greenland covered by our study. In (**a**) mean annual ice discharge (Gt a^−1^; symbol size) over 2000–2021 is shown along with the annual trend over this period (% a^−1^; colour code). The extents of the seven sectors considered are marked in addition. In (**b**) cumulative mean annual ice discharge (Gt a^−1^) over 2000–2021 summed up over the 489 peripheral tidewater glaciers is shown. Glaciers are sorted in descending order of ice discharge. The respective annual ice discharge trends (% a^−1^) of the glaciers are shown as colour code. The numbers of tidewater glaciers (upper x axis) needed to reach key percentages of total Greenland-wide ice discharge (right y axis) are indicated by dashed lines. The scatter plot in (**c**) shows annual ice discharge trends (% a^−1^) in relation to mean annual ice discharge (Gt a^−1^) of the 489 peripheral tidewater glaciers over 2000–2021. Means per sector are marked by named squares. Annual ice discharge trends (% a^−1^) are colour-coded while trend significance (*p* < 0.1) is indicated by bold symbol outlines. A linear fit to the data is shown as dashed line.

The decrease of ice discharge from the PTG over the latest two decades allows for the hypothesis of a differing evolution of ice dynamics at the PTG compared to what is known for the marine-terminating outlet glaciers of the GIS, and, in particular, a differing or even decoupled response to ocean thermal conditions^8,13^. In our study we work out to which extent this hypothesis holds true. We analyse if regional variability around Greenland has to be considered in this respect and how closely the evolution of ice discharge from the PTG is linked to ocean thermal forcing (OTF). In order to do so, we create an independent ice discharge estimate for the PTG, which (in contrast to the previous studies) strictly relies on a fully automatic and thus objectively reproducible methodology. Our study covers the period 2000–2021. For our analyses, we subdivide the PTG into seven sectors (N, NE, E, SE, S, W and NW; Fig. 1a) around Greenland, and report our results accordingly.

## Results

### Ice discharge at individual glaciers

We estimate ice discharge for 489 PTG which account for 94.5% of the PTG area considered (Methods, Fig. 1, Table 1). Ice discharge from these glaciers during the 2000–2021 period shows a mean annual rate of 5.40 ± 3.54 Gt a^−1^ (Fig. 1b). Mean ice discharge from individual glaciers is highly variable across Greenland and spans over seven orders of magnitude (Fig. 1a,c). It reaches 0.67 ± 0.13 Gt a^−1^ at the largest single contributor, which is Berlingske Bræ in NW Greenland (Fig. 1a). In addition to this outstanding glacier, the Greenland-wide sum of ice discharge from PTG is dominated by only a few other major contributors (Fig. 1b). A subset of 10 glaciers contributes ~ 50% to the Greenland-wide sum, while 82 are needed to reach ~ 90%. With approximately half of the PTG that are considered in our study (247), 99% of the Greenland-wide sum is accounted for. Annual ice discharge trends over 2000–2021 are likewise highly variable within a maximum range of ± 11% a^−1^ (Fig. 1). 173 (33.8%) of the glaciers considered show a significantly (*p* < 0.1) positive ice discharge trend and 77 (15.0%) a significantly (*p* < 0.1) negative one. We further found a clear relationship between absolute ice discharge amounts and their change over time: the smaller the ice discharge from a glacier, the more positive is its temporal trend (Fig. 1c). For the larger contributors (above ~ 0.01 Gt a^−1^), this even turns into a tendency towards increasingly negative ice discharge trends with further increasing absolute ice discharge amounts.

**Table 1:** Distribution of peripheral tidewater glaciers over seven sectors of Greenland (Fig. 1), and mean annual ice discharge over 2000–2021 as well as ice discharge and ocean thermal forcing trends per sector over this period.

Sector	Number of considered (covered) tidewater glaciers	Area (in km^2^) of considered (covered) tidewater glaciers	Annual ice discharge (Gt)	Annual ice discharge trend	Annual ocean thermal forcing trend
N	93 (84) *18.2% (17.2%)*	7,039 (6,555) *26.2% (25.8%)*	0.99 ± 0.34 *(18.3%)*	+ 1.1% *(not sign.)*	+ 0.9% (+ 0.02 K a^−1^) *(p* = *0.000002)*
NE	45 (42) *8.8% (8.6%)*	6,282 (6,245) *23.4% (24.6%)*	1.30 ± 1.15 *(24.1%)*	− 2.7% *(p* = *0.000014)*	+ 0.4% (+ 0.04 K a^−1^) *(p* = *0.000018)*
E	62 (62) *12.1% (12.7%)*	2,088 (2,088) *7.8% (8.2%)*	0.27 ± 0.11 *(5.0%)*	+ 1.9% *(p* = *0.0095)*	+ 0.3% (+ 0.04 K a^−1^) *(p* = *0.000006)*
SE	110 (109) *21.5% (22.3%)*	3,418 (3,405) *12.7% (13.4%)*	0.76 ± 0.48 *(14.1%)*	+ 0.7% *(not sign.)*	− 0.1% (− 0.002 K a^−1^) *(not sign.)*
S	143 (137) *27.9% (28.0%)*	4,187 (4,072) *15.6% (16.0%)*	1.14 ± 1.09 *(21.1%)*	− 0.1% *(not sign.)*	− 0.1% (− 0.004 K a^−1^) *(not sign.)*
W	36 (32) *7.0% (6.5%)*	2,209 (1,390) *8.2% (5.5%)*	0.08 ± 0.06 *(1.4%)*	− 0.4% *(not sign.)*	− 0.9% (− 0.03 K a^−1^) *(p* = *0.00025)*
NW	23 (23) *4.5% (4.7%)*	1,618 (1,618) *6.0% (6.4%)*	0.87 ± 0.33 *(16.0%)*	+ 1.4% *(p* = *0.028)*	+ 0.8% (+ 0.01 K a^−1^) *(p* = *0.00072)*
Total	512 (489)	26,841 (25,373)	5.40 ± 3.54	− 0.13% *(not sign.)*	+ 0.4% (+ 0.01 K a^−1^) *(p* = *0.0034)*

For each sector, numbers and areas of considered tidewater glaciers and the respective subsets that show sufficient data coverage for ice discharge calculations (in parentheses) are given. For numbers, areas of glaciers and ice discharge per sector, the shares in the respective Greenland-wide total are given in addition (lower line, in italics). Trends (Sen's slope) are expressed as percentage of mean annual ice discharge and ocean thermal forcing, respectively. They are given along with information regarding their statistical significance (*p* value of Mann–Kendall trend test, lower line, in italics). Absolute values of annual ocean thermal forcing trends are shown in addition (in parentheses).

### Regional variability of ice discharge

**Figure 2:**
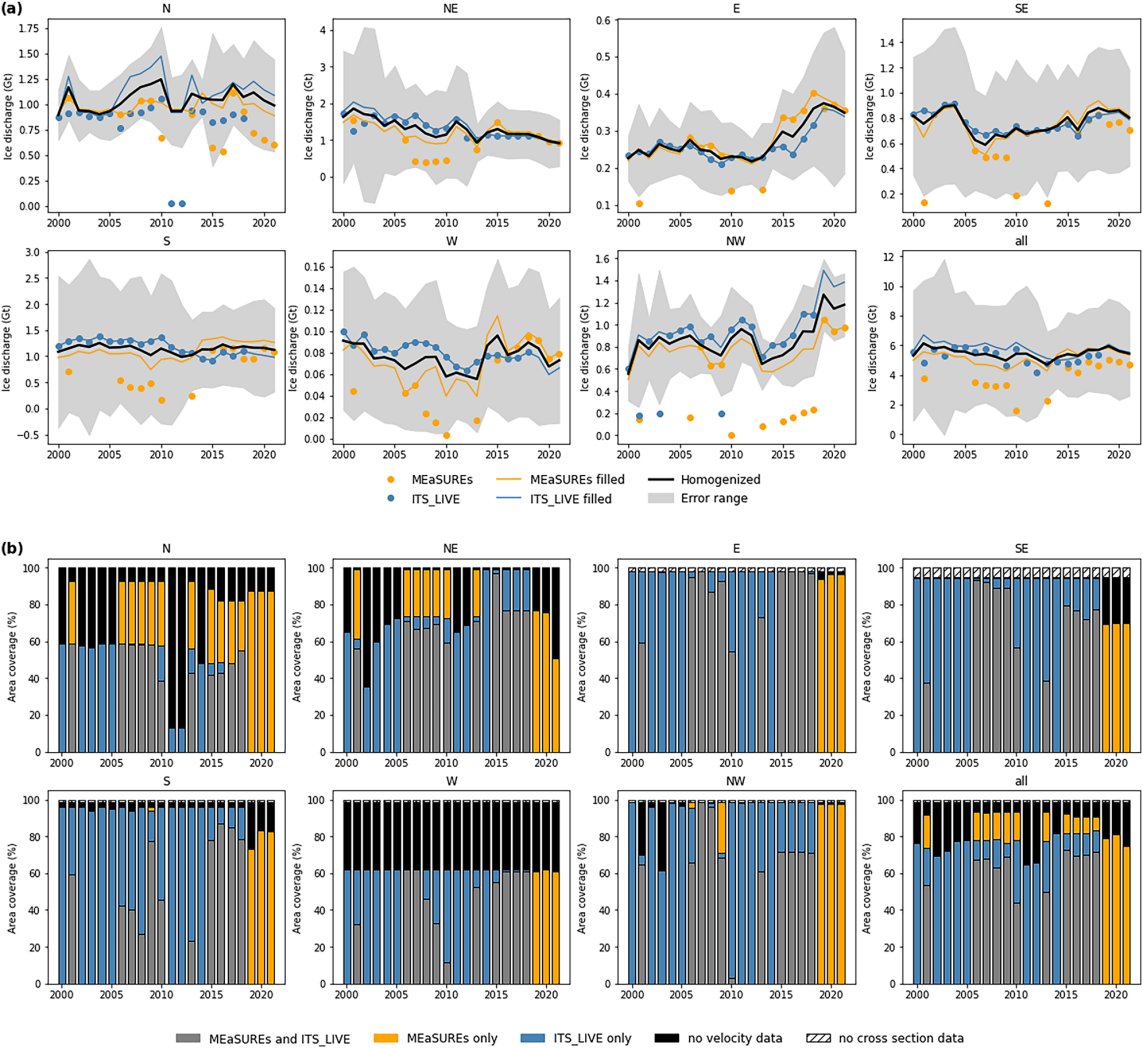
Time series of the ice discharge sum from the peripheral tidewater glaciers in each sector over 2000–2021 and coverage fractions of different datasets. In (**a**) annual ice discharge sums calculated from MEaSUREs and ITS_LIVE velocity data (colour-coded point symbols) are shown along with the respective gap-filled (cf. Methods) time series of annual ice discharge (colour-coded lines). Final, homogenized annual ice discharge (thick black line) is shown together with its uncertainty range (grey shading). In (**b**) the percentages of glacier area within each sector that are covered by either MEaSUREs or ITS_LIVE or both velocity datasets are shown. Percentages of glacier area for which an ice discharge calculation is not possible because of missing velocity or cross section data are shown in addition.

Unlike the distribution of ice discharge across the individual PTG, none of the seven sectors considered (Fig. 1a) plays a dominant role for the Greenland-wide sum of ice discharge from PTG on the first sight (Fig. 2a). However, considerable variability becomes evident when taking into account the uneven distribution of PTG across the different sectors (Table 1). The sums of ice discharge from the PTG in sectors S and NW were disproportionately large (Table 1) and thus of particular importance for the Greenland-wide sum. Ice discharge from the 143 PTG in sector S was 1.14 ± 1.09 Gt a^−1^ (21.1% of the Greenland-wide sum), even though they only account for 15.6% of the PTG area considered. The disproportionality of ice discharge from the 23 PTG in sector NW was even more pronounced at 0.87 ± 0.33 Gt a^−1^ (16.0% of the Greenland-wide sum) as they only cover 6.0% of the PTG area considered. However, this extraordinary role is because of the outstanding situation at Berlingske Bræ, which alone contributed 12.3% to the Greenland-wide sum of ice discharge from PTG (Fig. 1). Contributions from sector N and especially sector W, in contrast, were disproportionally small (Table 1). Sector N contributed 18.3% (0.99 ± 0.34 Gt a^−1^) despite an area share of 26.2% and sector W contributed only 1.4% (0.08 ± 0.06 Gt a^−1^) despite a distinctly higher area share of 8.2%. For the PTG in the other sectors, ice discharge contributions were in balance with the area they cover.

The temporal evolution of ice discharge from the PTG over 2000–2021 also shows distinct variability around Greenland (Fig. 2a, Table 1). Sector NW showed a significant (*p* < 0.05) increase of ice discharge at a trend of + 13.6% (+ 0.12 Gt) per decade over 2000–2021. The most pronounced relative increase of ice discharge over this period occurred in sector E, where we find a significant (*p* < 0.01) trend of + 19.5% (+ 0.05 Gt) per decade. In both sectors the increases of ice discharge, however, emerge not until the second half of the study period. In contrast to this, ice discharge in sector NE showed an almost continuous decrease over 2000–2021 along a significant (*p* < 0.001) trend of − 27.2% (− 0.35 Gt) per decade (Fig. 2a, Table 1). All other sectors did not show significant changes of ice discharge that form an unambiguous picture all over the two decades studied. Sector SE stands out with a decreasing ice discharge until 2007 and a significant (*p* < 0.001) trend of + 25.7% (+ 0.19 Gt) per decade afterwards (Fig. 2a). Apart from this spatially diverse pattern of ice discharge evolution, we find a general tendency towards decreasing ice discharges over all sectors that is evident from at least 2019 onwards (Fig. 2a). However, this period is far too short to draw any meaningful conclusions regarding temporal trends.

### Ocean thermal forcing

**Figure 3:**
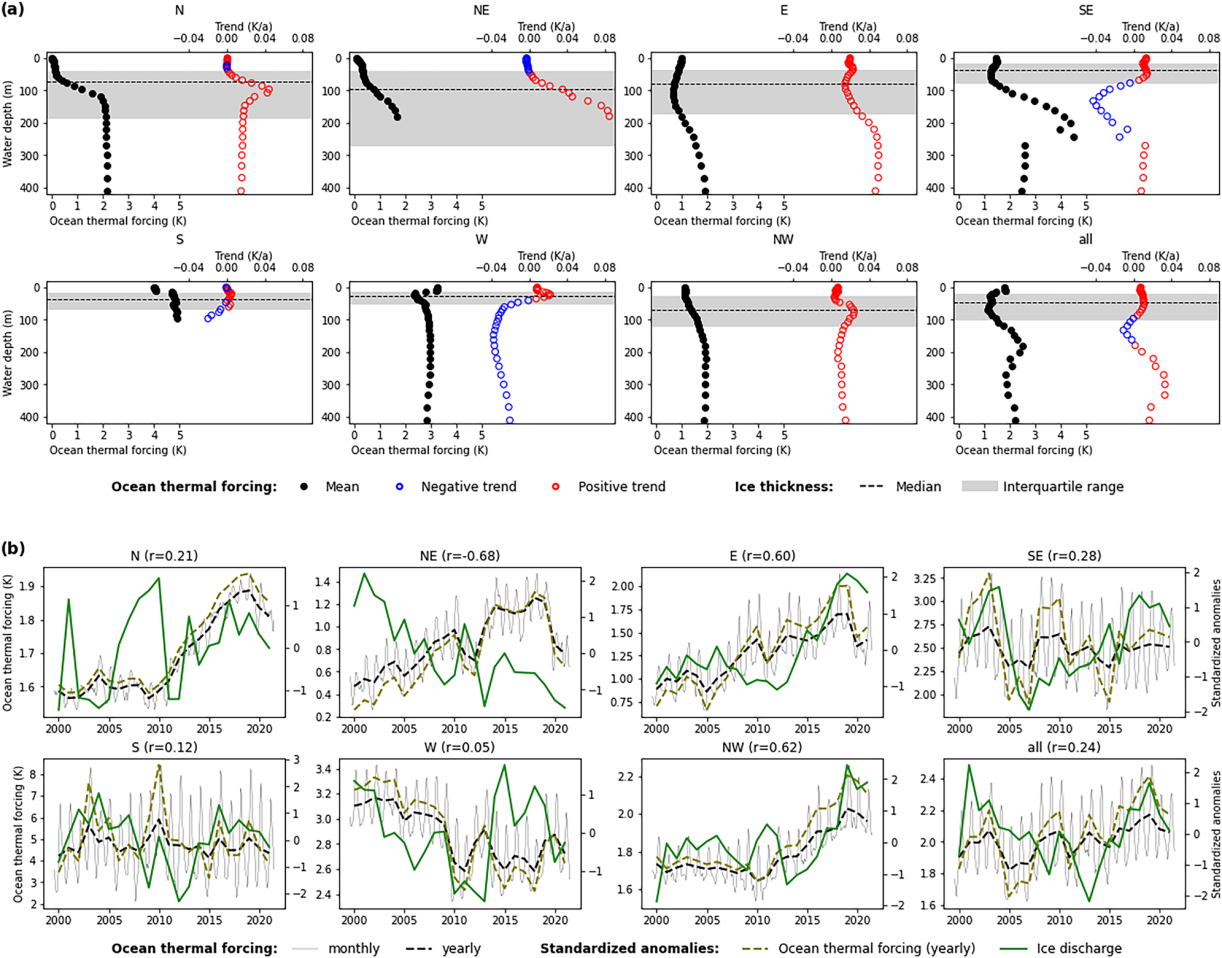
Mean ocean thermal forcing at the peripheral tidewater glaciers in each sector during 2000–2021 according to ORAS5 data. In (**a**) depth profiles of mean ocean thermal forcing during 2000–2021 are shown along with the respective temporal trends. The distribution of ice thickness at the fronts of the tidewater glaciers in each sector is indicated by median and interquartile range values. In (**b**) the time series of monthly and yearly ocean thermal forcing are displayed (left y axes) together with the standardized anomalies of yearly ocean thermal forcing (right y axes). Standardized anomalies of the homogenized annual ice discharge (cf. thick black line in Fig. 2a) are displayed for comparison. Pearson correlations between the standardized anomalies of ocean thermal forcing and ice discharge are indicated in parentheses at the top of each regional panel.

OTF shows a distinct variability around Greenland and across water depth at the PTG fronts (Fig. 3a). In sectors N and NE considerable variability with water depth is evident. Annual mean OTF is close to zero in the uppermost decameters of the ocean water column but increases with depth, before reaching > 2 K at deeper grounding line depths. OTF along the eastern Greenland coast is rather invariable down the water column, at least across most of the PTG grounding line depths in the respective sectors. OTF in sector E is ~ 1 K only. It increases to ~ 1.5 K in sector SE before jumping to ~ 4.8 K in sector S. Across sectors W and NW OTF again decreases with water depth and towards more northerly regions. In sector W, OTF at most of the PTG ground line depths is in the range 2.4–2.8 K, while in region NW only 1.1–1.6 K are reached.

The temporal evolution of OTF around Greenland over 2000–2021 reveals a clear pattern that ranges from a significant warming trend in the northern and northeastern regions over unchanged conditions in the southern regions towards a significant cooling in the West (Fig. 3b, Table 1). The strongest relative increase in OTF occurs in sector N (+ 9.4%/ + 0.16 K per decade), while showing a pronounced positive spike at ~ 100 m water depth, where the decadal trend reaches up to + 0.44 K (Fig. 3a). The relative increase of OTF in sector NW is comparably strong (+ 7.6%/ + 0.14 K per decade), and shows a similar but less pronounced spike at ~ 90 m water depth (Fig. 3a). Increases in OTF in sectors NE and E are smaller in relative but distinctly larger in absolute terms (+ 4.8%/ + 0.38 K per decade and + 2.9%/0.37 K per decade). Sector NE hereby stands out with an OTF trend that continuously increases with water depth (Fig. 3a). Further south along the eastern coast of Greenland (i.e. in sectors SE and S) ocean thermal forcing does not show a significant trend over 2000–2021 anymore. This departure from a regional ocean warming culminates in sector W, where OTF finally shows a significantly negative trend of − 8.7% (− 0.25 K) per decade when integrated over the top 700 m of the water column. However, across the grounding line depths of the PTG in this sector, the OTF trend shows a pronounced positive spike of + 9.4% (+ 0.22 K) per decade at ~ 23 m water depth which is similar to the spikes observed further north along the western coast (i.e. in sectors NW and N; Fig. 3a). As already for ice discharge, we also find a general tendency towards decreasing OTF from 2019 onwards (Fig. 3b) that exists in all sectors and thus independent of the spatially continuous pattern of OTF evolution described before. As for ice discharge, meaningful conclusions regarding temporal trends cannot be drawn here.

### Comparison of ice discharge and ocean thermal forcing

Ice discharge and OTF develop in comparable ways only in two of the seven sectors considered. In sectors E and NW, the significant positive trends of ice discharge and OTF (Table 1) lead to statistically significant correlations between the two variables of r = 0.60 (*p* < 0.01) in sector E and r = 0.62 (*p* < 0.01) in sector NW (Fig. 3b). The significant positive ice discharge trend that we observe for sector SE after 2007 does not coincide with a corresponding increase of OTF (Fig. 3b).

**Figure 4:**
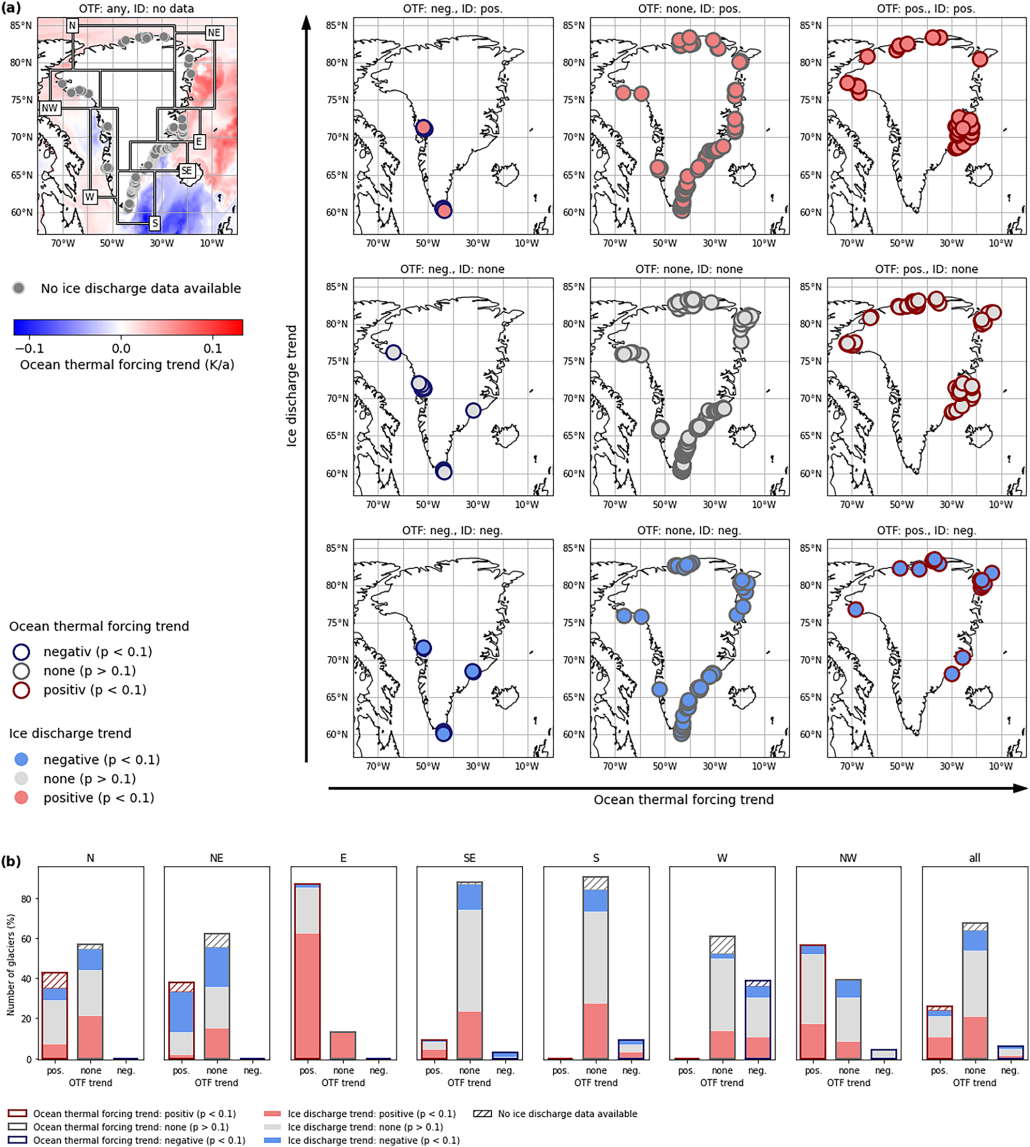
Comparison of ocean thermal forcing and ice discharge trends over 2000–2021 at the peripheral glaciers around Greenland. In (**a**) the 3 by 3 matrix on the right shows the locations of glaciers with a specific combination of ocean thermal forcing (OTF) and ice discharge (ID) trends (colour-coded). The panel in the upper left shows a map of 2000–2021 OTF trends around Greenland with the locations of glaciers for which an ice discharge calculation is not possible on top. In (**b**) the number of glaciers per sector (in % of the respective total number per sector) with the different combinations of ocean thermal forcing trends (colour-coded bar outlines) and ice discharge trends (colour-coded bar filling) are shown.

Accordingly, a strong cluster of individual PTG in sector E shows both, a significant (*p* < 0.1) positive OTF trend and a likewise significant positive ice discharge trend (Fig. 4a). A positive OTF trend occurs at 87% of the PTG in this sector. Among them, more than two thirds (72.2%) also experience positive ice discharge trends (Fig. 4b). The remaining 13% of the PTG in this sector do not show any OTF trend, but also show positive ice discharge trends without exception. The cluster of paired positive OTF and ice discharge trends extends southward into the very northern part of sector SE (Fig. 4a) where half of the PTG with positive OTF trends also show positive ice discharge trends (Fig. 4b). Further south, positive OTF trends are not observed any more (Fig. 4a).

In sector NW the picture is much more diverse and does not support the predominance of PTG that show positive OTF along with positive ice discharge trends (Fig. 4a). While such a predominance is suggested by the significant positive sector-wide trends of both variables (Fig. 3b), paired positive trends actually only occur at four of the 23 PTG in this sector (17.4%, Fig. 4b). Nine (39.1%) PTG show a positive OTF trend, but no positive ice discharge trend, and ten (43.5%) PTG either show no or a negative OTF trend (Fig. 4b).

Further along the northern coast, paired positive trends become even less frequent (Fig. 4b). The rather large share of 17.4% in sector NW diminishes over 7.5% in sector N to just one out of 45 PTG (2.2%) in sector NE. Simultaneously, the share of PTG that are affected by positive OTF trends and show a contrary negative ice discharge trend increases from just 4.3% in sector NW over 6.5% in sector N to 20.0% in sector NE (Fig. 4b). Accordingly, we find a significant negative correlation (r = − 0.68, *p* < 0.001, Fig. 3b) between the decreasing ice discharge (− 27.2% per decade) in sector NE and the concurrently increasing OTF along its coast (+ 4.5% or + 0.38 K per decade) (Table 1). In all other sectors around Greenland, statistically significant relations between sector-wide ice discharge and OTF do not exist (Fig. 3b).

## Discussion

### Role of ice discharge for regional and global glacier mass loss

**Figure 5:**
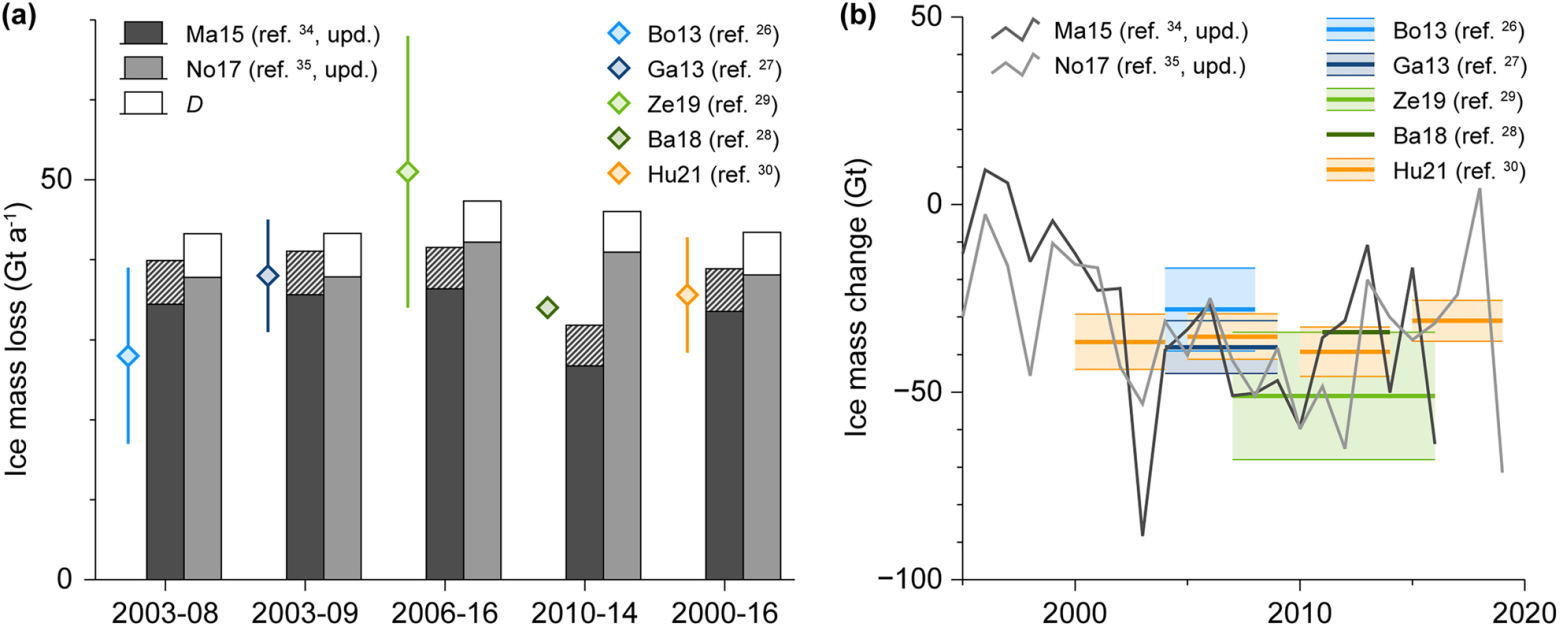
Comparison of previous ice mass loss estimates for Greenland's peripheral glaciers. Mean annual rates for different periods with independent, remotely-sensed estimates of overall ice mass loss (**a**) are shown along with the evolution of annual rates over recent decades (**b**). Ice mass loss due to modelled surface mass balance processes is shown as bars (**a**) and line graphs (**b**). Our ice discharge estimates *D* are shown as stacked bars for No17 and hatched area for Ma15, as the latter implicitly includes ice discharge (**a**). Remotely-sensed overall ice mass loss estimates are shown colour-coded and indicating uncertainties as given in the respective original studies.

Ice discharge from the PTG contributed significantly to the total mass loss of all peripheral glaciers (Fig. 5a). Based on our ice discharge calculation and available total mass loss estimates from previous studies^26–30^, the share of ice discharge from the PTG in the total mass loss from all peripheral glaciers ranges between 10.4% (2006–2016)^29^ and 20.1% (2003–2008)^26^. This substantial variability of the percentages does not reveal any temporal trend, but is explained by short-term surface mass balance variability and different methodologies in the total mass loss studies.

A more controversial picture emerges when considering our results together with the surface mass balance modelling studies that cover all of Greenland’s peripheral glaciers^34,35^ (Fig. 5b). Surface mass loss alone already exceeds several of the total ice mass loss estimates, which leaves little room for additional mass loss by ice discharge. As the order of magnitude of our results is supported by the other three available ice discharge estimates^36–38^, this suggests an overestimation of surface mass loss by the respective Greenland-wide surface mass balance modelling studies^34,35^. However, the slight increase of surface mass loss over large parts of our study period which is revealed in both modelling studies is implicitly supported by our results. Given the rather stable total ice mass change rates^30^ during 2000–2020 (Fig. 5b), this increase necessarily suggests a decreasing ice discharge as compensation, which is mirrored in our findings. Hence, in qualitative terms the different results form a coherent picture, but they do not in quantitative terms.

Also compared to the total mass loss from the GIS, the amount of ice discharge from the PTG is considerable. The GIS showed a total ice mass loss of 231 ± 28.7 Gt a^−1^ during 2002–2017^11^, while our estimate of ice discharge from the PTG during this period (5.37 ± 3.62 Gt a^−1^) corresponds to 2.3% of this number. Even globally, in the period 2006–2016, ice discharge from the PTG turns out to hold a share of 1.6% in the total mass loss from glaciers outside the two ice sheets^29^. When looking at the period 2000–2019 this share appears even larger (2.1%)^30^. Taken together, this indicates a substantial importance of the ice discharge from the PTG not only for Greenland and the Arctic, but also for global land ice mass loss.

### Regional distribution of ice discharge compared to main ice sheet

**Figure 6:**
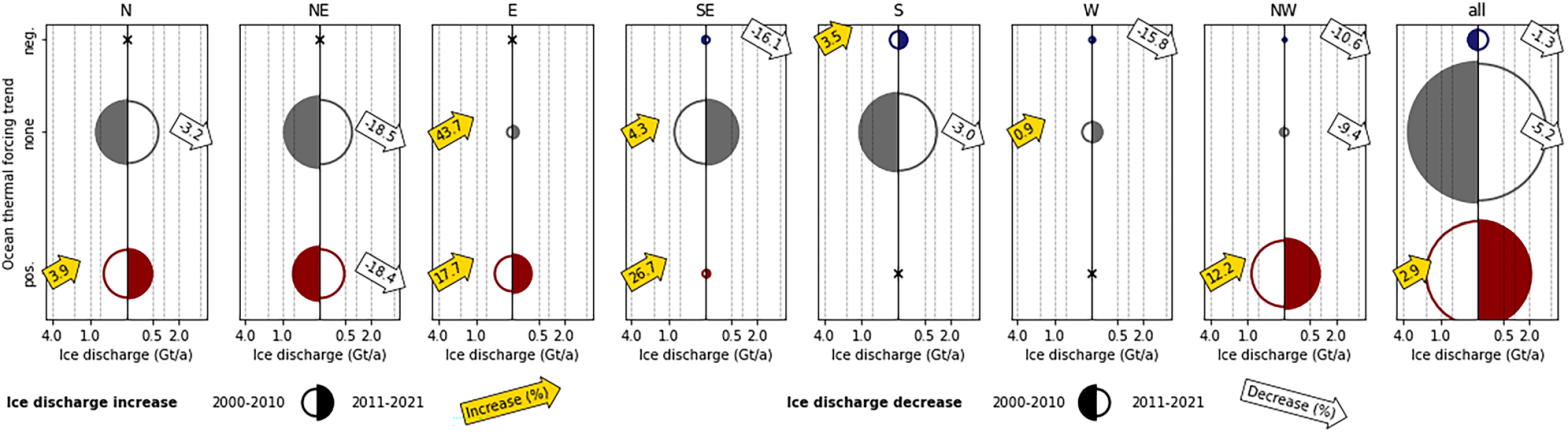
Ice discharge sums at peripheral tidewater glaciers in each sector, divided into different categories of trends of ocean thermal forcing in 2000–2010 and 2011–2021. Each panel represent a specific sector and shows ice discharge from tidewater glaciers with significantly (*p* < 0.1) positive (dark red), significantly negative (dark blue) and no significant (*p* > 0.1) trend in ocean thermal forcing. The left half of each circle represents 2000–2010 and the right half 2011–2021. The period with lesser ice discharge is displayed as open circle and the period with more ice discharge as filled circle. The increase or decrease in ice discharge between these two periods is shown in arrow symbols. The circle size indicates the respective amounts of mean annual ice discharge (Gt a^−1^), while the x axes (symmetrical around zero) quantify these amounts. If no glaciers in the respective category are present the circle symbol is replaced by an x.

Over the study period 2000–2021, especially the northern areas of the GIS have shown a rather continuous increase of ice discharge, while developments in the other areas are more heterogeneous in time^39^. This spatial pattern contrasts the development at the PTG. Ice discharge at the northern and southern most ends of Greenland (sectors N and S) is, in general, least variable. Changes of ice discharge between 2000–2010 and 2011–2021 lie always below 4% (Fig. 6). Changes of ice discharge in the sectors along the eastern and western coasts of Greenland are, in contrast, distinctly stronger.

The overall picture at the GIS has changed recently. Since 2016, the central-eastern and the south-eastern areas of the GIS show a steady increase of ice discharge, while stagnant or even decreasing ice discharge is evident across all other areas^39^. The recent increase of ice discharge from the central-eastern area of the GIS is mirrored by the predominance of increasing ice discharge from the PTG in sector E and in the northernmost part of sector SE, which occurs during the second half of your study period (Fig. 3a). In contrast, the increasing ice discharge from the south-eastern area of the GIS is not reflected in our results. For our sector S and the southern and central parts of sector SE, we find a rather stable ice discharge (Figs. 1a and 3a).

A particularly pronounced difference between the evolution of ice discharge from PTG and GIS in recent years seems to be evident in NW Greenland. While ice discharge from the respective area of the GIS has decreased almost continuously since 2017^39^, our results suggest a strong and continuous increase of ice discharge from the PTG in sector NW over the same period (Fig. 2a). However, this sector-wide increase is mainly based on the development of one single glacier. The region is home to the largest single contributor to the Greenland-wide sum of ice discharge from PTG. Berlingske Bræ (RGI 6.0^23^ ID RGI60-05.08041) in Steensby Land, Northwest Greenland (Fig. 1) is an outlet glacier of a local ice cap. It has shown considerable advance into Granville Fjord over most of the twentieth century, at least until 2009^40^, and partial thickening was observed across its lower tongue^41^. Corresponding to this behaviour, we found its ice discharge to have increased at a trend of + 13.8% (+ 0.09 Gt) per decade over 2000–2021.

Taken together, these findings indicate deviating evolution of ice discharge from PTG and GIS on a regional scale in most parts of Greenland over the study period. However, characteristic, sector-wide contrary evolution of ice discharge from PTG and GIS is only evident in NE Greenland. In this area of the GIS, ice discharge remained almost constant over the first half of the study period and started a slight but continuous increase afterwards^39^. The vast majority of PTG in sector NE on the other hand show a continuous decrease of ice discharge over the study period (Figs. 1a and 2a).

### Influence of ocean thermal forcing on ice discharge

The high spatial and temporal variability of OTF around Greenland (Figs. 3 and 4a) is caused by complex ocean dynamics, governing the exchange of heat between open ocean and shelf^42–46^. We do not intend to outline and explain the processes and reasons behind this variability, but exclusively focus on the impact that this variability has on ice discharge. Our findings suggest a heterogeneous and spatially confined influence of OTF on the ice discharge of individual PTG around Greenland. Sector-wide OTF and ice discharge trends only coincide in sectors E and NW (Fig. 3b). However, reasonable OTF influence on individual PTG is also suggested when considering the respective OTF trends and the corresponding changes of ice discharge together with the ice thicknesses at the glacier fronts (Figs. 4 and 6). Hereby, marked differences exist between increasing and decreasing OTF.

Ice discharge from PTG with positive OTF trends increases in all sectors except for sector NE (Fig. 6). This also imprints on the Greenland-wide ice discharge sums. The ice discharge sum from the 134 PTG (26.2% of all PTG considered) that show positive OTF trends increases by 2.9% between 2000–2010 and 2011–2021 (Fig. 6). On the first sight, this suggests a clear OTF influence. However, of these glaciers, only little more than one third (56, 10.9% of all PTG considered) also show positive ice discharge trends (Fig. 4b). The fact that total ice discharge increases nevertheless, is thus mainly because glaciers with rather large ice discharge are part of the 56-PTG double-positive subset. While the PTG of this subset show a mean annual ice discharge of 0.016 Gt over the study period, the mean of all other PTG amounts to just 0.011 Gt. It is, moreover, remarkable that the 56 PTG of this subset are characterized by a distinctly larger terminus ice thickness (121 ± 100 m compared to 74 ± 95 m; mean ± one sigma), which makes them more similar to the ice sheet outlets. On the one hand, these results clearly show that the influence of OTF that is present in most sectors is only felt by a limited number of individual PTG. But on the other hand, they also allow for the hypothesis that these individual PTG more directly react to OTF because of their larger terminus ice thickness and deeper grounding lines. Changes in effective OTF of the outlet glaciers of the GIS was found to be evident especially in deeper parts of the ocean water column and thus directly at the grounding lines^14,15^. Hence, it is reasonable to assume that similar processes exclusively occur at the largest of the PTG, while the shallow grounding line depth of the majority of the PTG prohibits their direct response to changes in OTF. In addition, the influence of upstream ice dynamics can be assumed to be relatively more important at PTG with thin termini than at those with thick termini and thus deeper grounding lines.

Ice discharge from PTG with negative OTF trends decreases in all sectors by at least 10% between 2000–2010 and 2011–2021 except for sector S, where it is suggested to increase by 3.5% (Fig. 6). As sector S provides by far the largest share in total ice discharge from PTG with a negative OTF trend (Fig. 6), this regionally confined increase is sufficient to reduce the overall Greenland-wide decrease.

And even if sector S provides by far the largest share in the sum of ice discharge from PTG with a negative OTF trend (Fig. 6), this picture also imprints on the respective Greenland-wide sum. Ice discharge from the 31 PTG (6.1% of all PTG considered) that show negative OTF trends decreases by 1.3% between 2000–2010 and 2011–2021 (Fig. 6). However, as only less than one fourth of these glaciers (7, 1.4% of all PTG considered) also show negative ice discharge trends (Fig. 4b), the behaviour of individual PTG is more likely to be the reason for this coincidence than a general OTF influence. Meaningful conclusions analogue to our before formulated hypothesis that an effective influence of changes in OTF on ice discharge is only evident at the largest of the PTG cannot be drawn in this case because of the small sample size.

As in the comparison of the regional evolution of ice discharge from PTG and GIS, sector NE also shows the most counterintuitive picture when relating ice discharge to OTF evolution. Over 2000–2021, ice discharge in this sector shows a statistically highly significant (*p* < 0.001) negative trend while OTF shows a statistically highly significant (*p* < 0.001) positive trend until 2019 (Fig. 3b). Considering the fact that the terminus ice thickness of the PTG in sector NE lies distinctly above average (181 ± 209 m compared to 71 ± 81 m in the other regions), and considering our hypothesis than an effective OTF influence on ice discharge especially occurs at the larger and thicker PTG, this leads to a clear but regionally confined contradiction to what happens in the rest of Greenland. We understand this apparent contradiction as an indication that OTF and ice discharge in this sector are part of a mechanism that is not resolved by our datasets, i.e., that the fjord circulation might lead to a different distribution of temperature compared to the other sectors. We can further rule out decreased melt as a reason for glacier slowdown as ERA5^47^ air temperature variability in sector NE shows a positive trend consistent with all other sectors. Ice discharge in sector NE is thus negatively correlated to air temperature (cf. supplementary Figure S4), as it is also to OTF. Parts of the high ice discharge sums in sector NE at the start of the study period might indeed be related to surge activity in the northwesterly basins of Flade Isblink ice cap at that time^48^, but also this cannot explain the rather homogeneous negative temporal trend (Fig. 3b). Hence, more research is needed to understand the reasons for the strongly deviating and counterintuitive behaviour of the PTG in sector NE.

## Methods

### General approach

In our analysis, we initially consider 512 PTG around Greenland. This number deviates from the entries in version 6.0 of the Randolph Glacier Inventory (RGI)^23^ or the inventory by Rastner et al.^22^ where 756 PTG are listed. This deviation results from our decision to leave out glaciers whose size we regard as insufficient for our analysis: In the gridded data-based parts of the workflow we have to deal with edge effects, i.e. errors at grid cells that extend across the glacier outlines and thus represent both glacier and off-glacier areas^49^. In order to avoid this, we reduce the area of the polygons of all 756 glaciers in the RGI 6.0 database by applying a 500 m buffer along the glacier outlines. This limits the number of PTG to 507, as 249 glacier polygons disappear. The remaining deviation originates from Flade Isblink ice cap, the largest of all PTG. In the RGI 6.0, Flade Isblink is included as one single tidewater glacier entity (RGI ID: RGI60-05.10315). However, the ice cap consists of different basins with varying flow directions, including six active tidewater glaciers^48,50^. We consider these basins separately instead of accounting for the whole ice cap as one single tidewater glacier (Supplementary Fig. S1).

In our analysis, we regard ice discharge as the flux of glacier ice through a cross section at the terminus of a tidewater glacier^51^. The discharged ice volume can be calculated as the product of the area of this cross section, i.e. of the flux gate, and the ice flow velocity through this flux gate, averaged vertically and horizontally^5,24,25,52^. By doing so, we estimate volume and mass of the ice discharge for the period 2000–2021.

We approximate the shape of the flux gate of each PTG by idealizing it as a rectangle, with its cross section made up of terminus width and terminus ice thickness. Data of both are computed by employing preprocessing routines of the Open Global Glacier Model (OGGM)^53^ analog to a previous Greenland-wide modelling study^50^. We cannot obtain terminus ice thicknesses for all PTG due to voids in the dataset or OGGM preprocessing errors. For the remaining PTG, we estimate terminus ice thickness by employing a regression forest model (see below for details). Data of surface ice flow velocity are derived from three different remote sensing-based datasets^54–56^. From surface ice flow velocity, we calculate vertically and horizontally averaged ice flow velocity through the flux gates of the PTG.

Because of permanent voids in the surface ice flow velocity datasets, our ice discharge calculation finally covers 489 of the 512 PTG considered (Table 1, Figs. 2b and 3b). This means we account for a PTG area of 25,373 km^2^ (Table 1) which equals 93.3% of the area of all PTG listed in the RGI 6.0 when corrected for number and size of the six individual tidewater basins of Flade Isblink ice cap. For these 489 PTG we then finally compare ice discharge to contemporaneous OTF, i.e. the difference between the actual water temperature and the salt and pressure dependent freezing point temperature at the glacier fronts, which we calculate on the basis of ORAS5 ocean reanalysis data^57^.

### Flux gate dimensions

For determining the idealized rectangular flux gate cross sections of the PTG (each made up of terminus width ${w}_{i}$ and terminus ice thickness ${h}_{i}$), we use two different methods. Terminus width (${w}_{i}$) for 493 PTG is computed on the basis of RGI 6.0 glacier outlines and gap-filled ArcticDEM^58^ elevation data using preprocessing routines of the OGGM^53^. For the remaining 19 PTG, preprocessing errors prevent the computation of terminus width. For 319 PTG with available terminus width, also terminus ice thickness (${h}_{i}$) is computed with the OGGM preprocessing routines, but using the global gridded ice thickness dataset of Millan et al.^59^ as input. With respect to tidewater glacier fronts, this dataset is superior to other global ice thickness datasets as it does not tend to underestimate close-to-terminus ice thicknesses as the other global gridded ice thickness datasets usually do^60^. Hence, it facilitates the straightforward extraction of more realistic terminus ice thicknesses. For the remaining 174 PTG with available terminus width, voids in the ice thickness grids or OGGM preprocessing errors prevent the extraction of terminus ice thickness from the dataset of Millan et al.^59^. For these glaciers, we estimate the terminus ice thicknesses (${h}_{i}$) using a regression forest model^61^ with 64 individual trees. The model is developed in Orange (version 3.34; https://orangedatamining.com), a Python-based data mining software, and trained with data of the 319 glaciers for which terminus ice thickness is available from the dataset of Millan et al.^59^. Our estimation is based on six glacier-specific variables that were readily available for each PTG either from the RGI 6.0^23^ (surface area, maximum and median surface elevation, central latitude) or from OGGM preprocessing (surface slope at the terminus, terminus width). The regression forest is grown by adding trees to the model until any further increase of the number of trees does not any more lead to further continuous increase or even any considerable change in model performance. This point is reached at a number of 64 trees (Supplementary Fig. S2). Thus, the number of trees in the regression forest model is determined as a trade-off between optimized model performance and minimized model complexity, which avoids overfitting^61^. At the stage of 64 trees, estimated ice thicknesses show a root mean square error (RMSE) of 61.5 m and a correlation of 0.81 (R^2^ = 0.66) compared to the ice thicknesses derived from Millan et al.^59^.

We rely on an extensive model usage for the computation of terminus ice thickness as it is the only feasible way to derive the required data in a fully automated and reproduceable way. Even the Greenland-wide ice thickness dataset BedMachine v3 lacks coverage at most of the PTG^25^.

### Ice flow velocity

For determining the mean annual vertically and horizontally averaged ice flow velocities through the flux gates (${v}_{i}$), we rely on own calculations on the basis of readily available surface ice flow velocity datasets^54–56^. We first extract mean surface ice flow velocity at the front of each PTG (${\overline{v}}_{surf,i}$) and then transfer it to depth-averaged ice flow velocity through the respective flux gate (${v}_{i}$). For this transfer, we consider the fact that depth-averaged ice flow velocity can roughly be approximated as a weighted mean of surface and basal flow velocities, with the former contributing 80% and the latter 20%^52^. This implies that depth-averaged ice flow velocity is confined to the range 80–100% of surface ice flow velocity, depending on the level of basal sliding under the glacier. We mediate between the two extreme values and calculate ${v}_{i}$ as

1 $${v}_{i}=0.9\times {\overline{v}}_{surf,i}.$$

Our analysis uses estimates of ${\overline{v}}_{surf,i}$ and thus of ${v}_{i}$ from the period 2000–2021 based on two different dataset families. Estimates of ${\overline{v}}_{surf,i}$ for the seven winter seasons 2000/2001, 2005–2010 and 2012/2013 are derived from 500 m resolution grids of the dataset MEaSUREs Greenland Ice Sheet Velocity Map from InSAR Data, Version 2^54^. Estimates for the seven winter seasons 2014–2021 are derived from 200 m resolution grids of the dataset MEaSUREs Greenland Quarterly Ice Sheet Velocity Mosaics from SAR and Landsat, Versions 3 and 4^55^. These two datasets complement each other and are used together as the first dataset family (we term it MEaSUREs). Annual estimates of ${\overline{v}}_{surf,i}$ for the whole period are derived from 240 m resolution grids of the ITS_LIVE dataset^56^. They form the second dataset family (we term it ITS_LIVE).

For estimating ${\overline{v}}_{surf,i}$, we calculate the mean surface ice flow velocity across the ${n}_{i}$ lowermost grid cells of each PTG inside its modified (500 m buffer) RGI 6.0 glacier outline^23^. These grid cells are meant to represent an idealized two-row stripe along the glacier front. Hence, ${n}_{i}$ is calculated on the basis of the terminus width of the respective glacier (${w}_{i}$) and the spatial resolution ($sr$) of the respective velocity dataset according to

2 $${n}_{i}=round\left(\frac{{w}_{i}}{sr}\times 2\right).$$

The involved surface elevation grids are resampled (to $sr$) versions of the same gap-filled ArcticDEM^58^ digital elevation model that we also use in the computation of terminus width.

We chose this approach for two different reasons. First, it facilitates an objective determination of the number of grid cells involved in the estimation of ${\overline{v}}_{surf,i}$ as it exclusively relies on glacier specific information. Alternative approaches like area- or elevation-related zoning of the grid cells involved would include an arbitrary selection of the applied threshold that imprints on the results derived. Second, we chose this approach in favour of an area- or elevation-related threshold in order to exclusively stick to glacier areas close to the terminus. By doing so, we avoid the inclusion of extensive, slower-flowing glacier areas that lie far away from the respective termini on long and flat tidewater glacier tongues.

### Ice discharge

For each PTG $i$ and year $y$, we calculate the mass of ice discharge (${D}_{i,y}$) from terminus width (${w}_{i}$) and terminus ice thickness (${h}_{i}$) at the flux gate and from time-varying, vertically and horizontally averaged annual ice flow velocity through this flux gate (${v}_{i,y}$) by assuming a constant glacier ice density of 917 kg m^−3^ according to

3 $${D}_{i,y}=0.917\times {w}_{i}\times {h}_{i}\times {v}_{i,y}.$$

This is done with both of the underlying velocity datasets, i.e. MEaSUREs velocity data (${D}_{i,y}^{M}$) and ITS_LIVE velocity data (${D}_{i,y}^{IL}$). The final, homogenized ice discharge estimate for each PTG and year is then calculated as the mean of these two estimates ($\overline{{D }_{i,y}}$). However, as the coverage of ${D}_{i,y}^{M}$ and ${D}_{i,y}^{IL}$ is inhomogeneous in both space and time (Fig. 2b), a two-way gap-filling procedure has to be applied first. This procedure assumes and exploits systematic relations between MEaSUREs and ITS_LIVE velocity data. We calculate linear fits (assuming an intercept of zero) between all ${D}_{i,y}^{M}$ and ${D}_{i,y}^{IL}$ that simultaneously exist for a specific PTG and year. This is done separately for each of the seven regions around Greenland (Supplementary Fig. S3). Two different linear fits are calculated for each region, i.e. one on the basis of ice discharge estimates for years within the period 2000–2013 and one for years within the period 2014–2021. This is done in order to account for a potentially differing behaviour of the two datasets within the MEaSUREs dataset family. Using these fits, we perform a two-way gap filling of the ${D}_{i,y}^{M}$ and ${D}_{i,y}^{IL}$ time series until the full 2000–2021 period is covered.

### Ocean thermal forcing

For each PTG, we calculate OTF using monthly mean data of potential temperature (${T}_{pot}$) and absolute salinity (${S}_{A}$) that were obtained from the ORAS5 ocean reanalysis dataset. This dataset shows an eddy-permitting horizontal resolution of 0.25° and a near-surface vertical resolution of 1 m that decreases with water depth. All calculations are performed using the *GSW-python* package^63^, which is a *Python* implementation of the Gibbs SeaWater (GSW) Oceanographic Toolbox of TEOS-10 (Thermodynamic Equation Of Seawater – 2010^64^). Based on ${T}_{pot}$ and ${S}_{A}$ we first calculate depth varying in situ and freezing point temperatures and then OTF as the difference between the two. The annual mean OTF used in our analysis is finally calculated from the respective monthly values. For calculation of annual mean OTF at each PTG we finally employ a depth-weighted mean over the water column down to 700 m depth at the ten horizontal grid locations that lie closest to the respective PTG front.

### Uncertainties

For each PTG $i$ and year $y$, we calculate the uncertainties of annual ice discharge (${U}_{\overline{D },i,y}$) from the individual uncertainties of the three multiplicands that determine ice discharge (${w}_{i}$, ${h}_{i}$ and ${v}_{i,y}$), following standard error propagation rules as

4 $${U}_{\overline{D },i,y}=\overline{{D }_{i,y}}\times \left(\frac{{U}_{w,i}}{{w}_{i}}+\frac{{U}_{h,i}}{{h}_{i}}+\frac{{U}_{v,i,y}}{{v}_{i,y}}\right).$$

Hereby, the uncertainties of terminus width (${U}_{w,i}$), terminus ice thickness (${U}_{h,i}$) and of mean annual vertically and horizontally averaged ice flow velocity through the flux gate (${U}_{v,i,y}$) vary from glacier to glacier, the latter also varies from year to year.

For determining ${U}_{w,i}$ of each PTG, we rely on the same method as we do to derive terminus widths ${w}_{i}$. The OGGM preprocessing routines not only deliver ${w}_{i}$ for the terminus itself, but also for regularly spaced cross sections along the central flow line of the glacier. We here use the standard deviation of the five lowermost cross section widths as our measure for terminus width uncertainty.

Determining ${U}_{h}$ requires the consideration of two different cases. For the 319 PTG for which terminus ice thickness ${h}_{i}$ is extracted from the dataset of Millan et al.^59^ by the OGGM preprocessing routines, uncertainty ${U}_{h}$ is analogously extracted by these routines from the ice thickness error estimates in the dataset of Millan et al.^59^. For the remaining 174 PTG for which ${h}_{i}$ is estimated using the regression forest model, we consider an uncertainty ${U}_{h}=61.5 m$, which equals the model's RMSE.

Determining ${U}_{v,i}$ for each PTG requires the consideration of the uncertainties of surface ice flow velocities at the glacier fronts (${U}_{\overline{v},surf,i}$) and of the influence of our assumptions during calculation of vertically-averaged ice flow velocities (Eq. 1). We derive glacier specific ${U}_{\overline{v},surf,i}$ from the error grids included in the MEaSUREs and ITS_LIVE surface ice flow velocity datasets. This is done similar to deriving the surface ice flow velocities at the glacier fronts (${\overline{v}}_{surf,i}$) by calculating the mean across the ${n}_{i}$ lowermost grid cells of each PTG (Eq. 2). Besides that, our usage of a mediating value (0.9, i.e. 90%) in the transfer from surface to vertically-averaged ice flow velocity (Eq. 1) introduces an additional uncertainty amounting to 10% of ${\overline{v}}_{surf,i}$. Consequently, and following quadratic error propagation rules, we calculate ${U}_{v,i}$ as

5 $${U}_{v,i}=\sqrt{{\left({U}_{\overline{v},surf,i}\right)}^{2}+{\left({0.1\times \overline{v}}_{surf,i}\right)}^{2}}.$$

These uncertainty estimates for ice discharge from the individual PTG are partly based on either MEaSUREs or ITS_LIVE annual ice flow velocity data. Hence and equal to ice discharge itself, these uncertainty estimates are not available for all the years of the study period. For our per-sector ice discharge results, we calculate a gap-filled and homogenized combination of both ice discharge estimates (see above). A similar procedure is not feasible for the uncertainty estimates and we rely on an alternative extrapolation method for the calculation of meaningful sector-wide uncertainties instead.

We assume that the relation between sector-wide annual ice discharge as it is calculated from either MEaSUREs or ITS_LIVE ice flow velocity data (point symbols in Fig. 2a) and the respective annual uncertainty also holds for the final, gap-filled and homogenized ice discharge time series. Consequently, we calculate the total annual ice discharge uncertainty (${U}_{\overline{D },r,y}$) of sector $s$ and year $y$ as

6 $${U}_{\overline{D },s,y}=\overline{{D }_{s,y}}\times \frac{1}{2}\left(\frac{{U}_{D,s,y}^{M}}{{D}_{s,y}^{M}}+\frac{{U}_{D,s,y}^{IL}}{{D}_{s,y}^{IL}}\right).$$

Additional, but unquantifiable uncertainty has to be considered with respect to our comparative analysis between ice discharge and OTF. Ice discharge from the PTG and the exchange of heat between ice and ocean at the glacier fronts occurs in fjords that are much too small to be resolved in global or even regional ocean models, thus ocean conditions provided in the ORAS5 dataset might not represent the real conditions next to the glacier^65^. This is even more true the smaller and more complex the fjords become. However, we are confident that the trends in OTF very likely propagate into these fjords in some form, so we do not feel this unquantifiable uncertainty materially affects our conclusions regarding the relationship between ice discharge and OTF.

### Supplementary Information

Supplementary Table S1.

Supplementary Figures.

## Data Availability

The MEaSUREs and ITS_LIVE surface ice flow velocity grids are accessible from the NASA Distributed Active Archive Center (DAAC) at NSIDC (https://nsidc.org/data/measures/gimp) and from the dedicated web app (https://nsidc.org/apps/itslive/), respectively. The ArcticDEM 100 m grid is available from the Polar Geospatial Center (PGC) at the University of Minnesota (https://www.pgc.umn.edu/data/arcticdem). ORAS5 data are accessible from the Copernicus Climate Data Store (https://cds.climate.copernicus.eu). For each of the seven sectors considered in this study, data of annual ice discharge including the associated uncertainty and of annual depth averaged OTF over the upper 700 m of the ocean water column are provided in the supplementary materials (Table S1).
