# Supplementary material for: Heterogeneous impacts of ocean thermal forcing on ice discharge from Greenland's peripheral tidewater glaciers over 2000–2021
Source: Sci Rep. 2024 May 17;14:11316. doi: 10.1038/s41598-024-61930-6 (PMC11101662; doi:10.1038/s41598-024-61930-6)

Supplementary Figures

to

Heterogeneous impacts of ocean thermal forcing on ice discharge from Greenland's peripheral tidewater glaciers over 2000-2021

by

Marco Möller, Beatriz Recinos, Philipp Rastner & Ben Marzeion

Fig. S1: Tidewater basins (orange outlines) of Flade Isblink ice cap (red outline). From the six tidewater basins, only four are considered in this study. The other two feature floating shelf ice across their marine-terminating frontal parts[48]. This prohibits a reliable handling in the Open Global Glacier Model (OGGM), resulting in missing information about their flux gate cross sections. The large seemingly marine-terminating ice lobes in the East of the ice cap do not show any signs of significant ice flow[48,50] and were thus not regarded as tidewater basins here. The six basins were numbered clockwise in order to include them in Extended Data Table 1, following the naming convention RGI60-05.10315_X. This combines the Randolph Glacier Inventory 6.0 ID of the ice cap[23] with the number of the respective basin ('X').

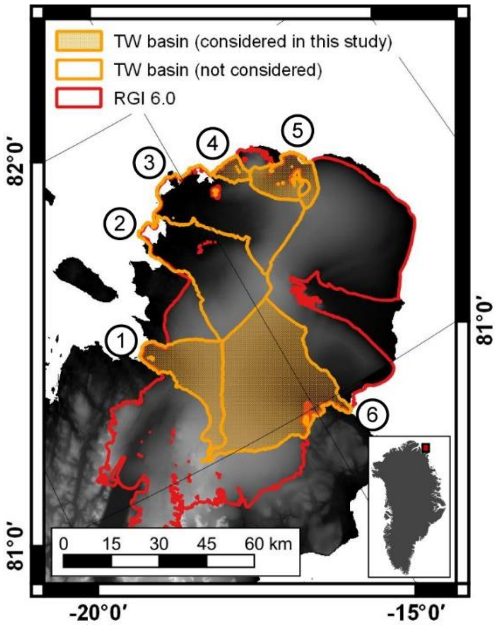

Fig. S2: Development of the performance of the regression forest model for estimation of terminus ice thicknesses with increasing number of trees. The root mean square errors (RMSE) and the R^2 served as performance measures *p*. They were calculated on the basis of the 319 peripheral tidewater glaciers that were available from Millan et al.[59] and served as the training dataset for the model. The calculation was done in an automated random 10-folds cross validation (within the applied Python-based data mining software Orange). Beyond 64 trees (shaded background), no significant increase of model performance occurred.

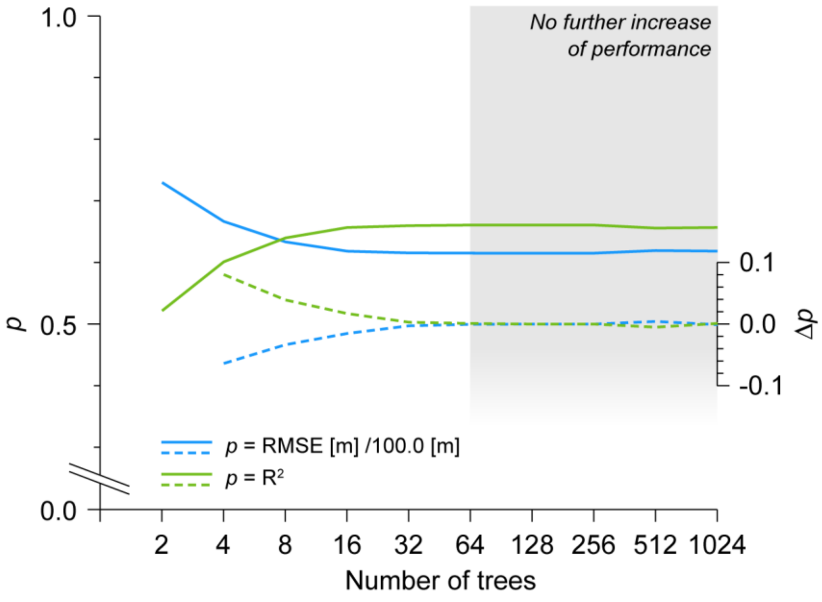

Fig. S3: Comparison of ice discharge (in Gt) at individual peripheral tidewater glaciers in the seven sectors (name indicated on top of each panel) as calculated with either MEaSUREs or ITS_LIVE ice flow velocity data. The scatter plots show yearly ice discharge values from two different periods (cf. Methods of main article for details). For the ice discharge data of each period a linear fit with a zero intercept is shown (colour code). The slopes of the linear fits are given at the lower right of the figure.

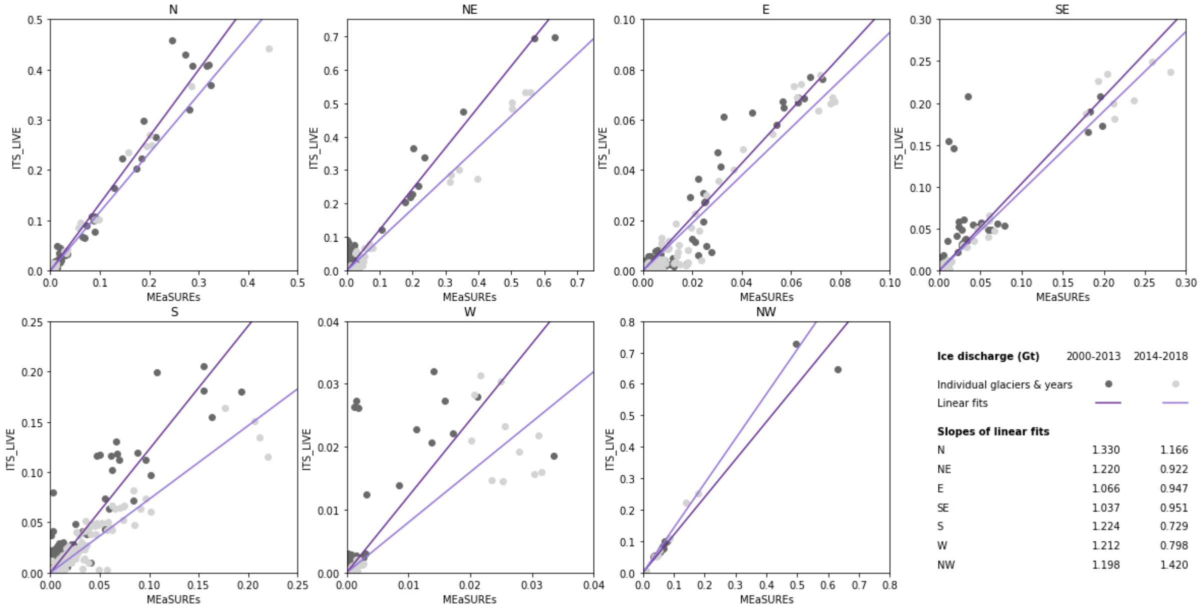

Fig. S4: Comparison of correlations between annual time series of sector-wide ice discharge and ocean thermal forcing (ID/OTF) and sector-wide ice discharge and positive degree days (ID/PDD). Ice discharge (ID) is calculated as the sum over all peripheral tidewater glaciers that are considered in the respective sector. Ocean thermal forcing (OTF) is calculated from ORAS5 data as outlined in the methods section of the main article. Positive degree days (PDD) are calculated from ERA5 six-hourly (00:00, 06:00, 12:00, 18:00) 2 m air temperature data[47] from the grid point closest to the respective glacier terminus. The comparison shows that correlations with OTF are distinctly larger (or at least similar) than correlations with PDD. Only the sector W differs from this general pattern.

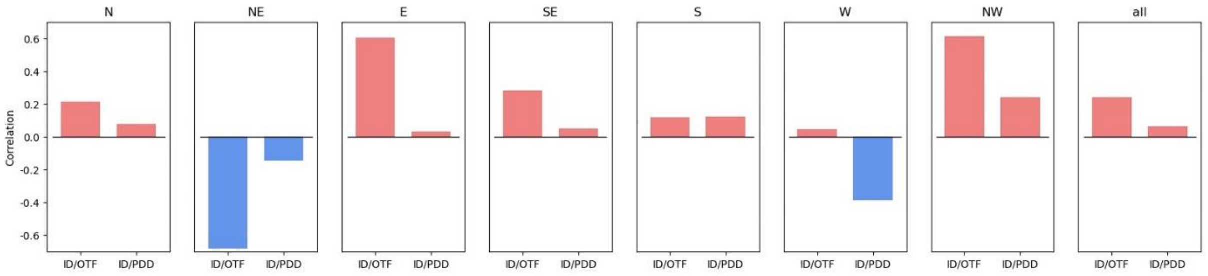

Supplement: Supplementary file 2 — Supplementary Figures. [file 41598_2024_61930_MOESM2_ESM.pdf]
